# Pediatric Acute Myeloid Leukemia (AML): From Genes to Models Toward Targeted Therapeutic Intervention

**DOI:** 10.3389/fped.2019.00401

**Published:** 2019-10-15

**Authors:** Thomas Mercher, Juerg Schwaller

**Affiliations:** ^1^INSERM U1170, Equipe Labellisée Ligue Contre le Cancer, Gustave Roussy Institute, Université Paris Diderot, Université Paris-Sud, Villejuif, France; ^2^Department of Biomedicine, University Children's Hospital Beider Basel (UKBB), University of Basel, Basel, Switzerland

**Keywords:** pediatric AML, genomic landscape, mouse models, fusion oncogene, therapeutic targeting, UKBB

## Abstract

This review aims to provide an overview of the current knowledge of the genetic lesions driving pediatric acute myeloid leukemia (AML), emerging biological concepts, and strategies for therapeutic intervention. Hereby, we focus on lesions that preferentially or exclusively occur in pediatric patients and molecular markers of aggressive disease with often poor outcome including fusion oncogenes that involve epigenetic regulators like KMT2A, NUP98, or CBFA2T3, respectively. Functional studies were able to demonstrate cooperation with signaling mutations leading to constitutive activation of FLT3 or the RAS signal transduction pathways. We discuss the issues faced to faithfully model pediatric acute leukemia in mice. Emerging experimental evidence suggests that the disease phenotype is dependent on the appropriate expression and activity of the driver fusion oncogenes during a particular window of opportunity during fetal development. We also highlight biochemical studies that deciphered some molecular mechanisms of malignant transformation by KMT2A, NUP98, and CBFA2T3 fusions, which, in some instances, allowed the development of small molecules with potent anti-leukemic activities in preclinical models (e.g., inhibitors of the KMT2A–MENIN interaction). Finally, we discuss other potential therapeutic strategies that not only target driver fusion-controlled signals but also interfere with the transformed cell state either by exploiting the primed apoptosis or vulnerable metabolic states or by increasing tumor cell recognition and elimination by the immune system.

## Genomic Landscape of Pediatric AML

### From Cytogenetics to Next-Generation Sequencing

Molecular hematology–oncology starting in the 1970s of the last century was heavily influenced by the pioneering work of Janet Rowley and others that used conventional cytogenetics followed by the upcoming recombinant DNA technology to show that, in addition to other structural lesions, balanced chromosomal translocations frequently lead to expression of fusion genes (1). Following these developments, the classification of leukemia evolved from a morphology-based classification to the progressive, and still ongoing, inclusion of genetic-based criteria (2, 3). During the last decade, high-throughput sequencing technologies (often referred to as next-generation sequencing, NGS) have facilitated the establishment of almost complete maps of the genomic landscape of leukemic cells in acute myeloid leukemia (AML) patients (4). In landmark studies by Timothy Ley and members of the Cancer Genome Atlas Research Network, the genome of a single AML patient was obtained in 2008. They later sequenced the whole genome of 24 selected AML cases but also the exomes of the progeny of hematopoietic stem and progenitor cells (HSPCs) taken from seven healthy individuals of different age (5, 6). Subsequently, they characterized the genomes of 200 clinically annotated adult cases of *de novo* AML either by whole-genome sequencing or exome sequencing along with RNA, miRNA sequencing, and DNA methylation analysis (7). Together with previous genetic and functional studies, several important observations can be highlighted. Firstly, the mutational rate of AML cells is lower than for most other cancers. Secondly, almost all samples had at least one mutation in genes of nine different categories [transcription factor fusions, nucleophosmin (NPM1), tumor suppressors, DNA-methylation-related genes, signaling mediators, chromatin modifiers, myeloid transcription factors, cohesin genes and spliceosome complex]. Thirdly, recurrent patterns of co-existence suggested functional cooperation as previously reported for transcription factor fusions/mutations [often referred to as “class II mutations”] and signaling mutations in tyrosine kinases or RAS-type GTPase (RAS) [often referred to as “class I mutations”] but also novel mutations targeting epigenetic regulators such as DNA methyltransferase 3a (DNMT3A) and isocitrate dehydrogenase (IDH)1/2 became apparent. Together with functional studies, these associations suggest that as little as two mutations in different categories might be sufficient to initiate leukemogenesis. Finally, the data obtained from healthy individuals suggested that the HSC compartment accumulates about 10–15 single-nucleotide variants every year.

**Figure 1:**
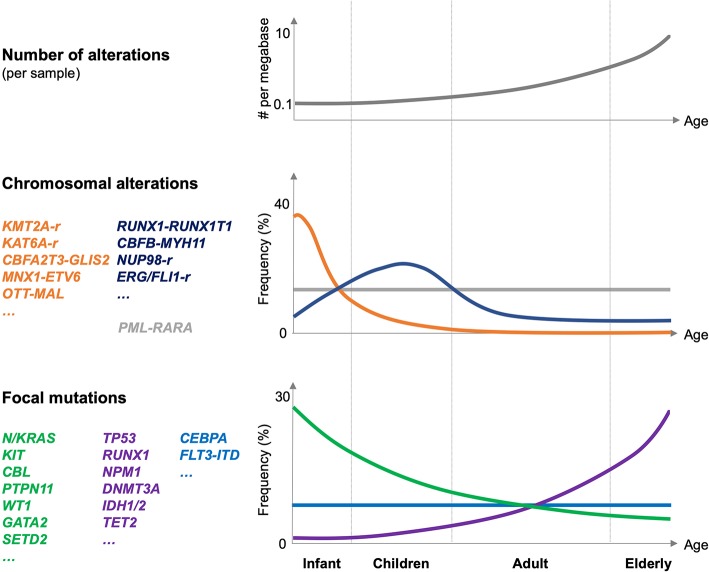
Genetic alterations in pediatric AML. Schematic illustration of the number of genetic alterations (per AML sample, **top panel**), frequency, and type of chromosomal alteration **(middle panel)**, and frequency and type of focal mutations in pediatric AML related to different age groups (infant, children, adults, and elderly; **lower panel**). Frequencies are indicated globally for the entire group of indicated mutations. The figure is mostly based on Bolouri et al. (8).

Over a decade later, the Children's Oncology Group (COG)–National Cancer Institute (NCI) TARGET AML initiative was able to characterize the genomic landscape of almost 1,000 pediatric AML patients by whole genome sequencing of samples from 197 and targeted sequencing of tumor cells from 800 patients (8). This extensive effort revealed similarities but also important differences between adult and pediatric AML. First, the overall somatic mutation frequency in pediatric AML is lower than that in adult patients. Notably, the mutational burden increases with age, with fusions and focal copy number aberrations being more common in younger patients, whereas smaller sequence variants are more frequent in older individuals. Second, pediatric AML patients with fusions involving transcriptional regulators like lysine methyltransferase 2A (KMT2A), CBFA2/RUNX1 translocation partner 3 (CBFA2T3), or motor neuron and pancreas homeobox 1 (MNX1) tend to have few additional mutations and were associated with a particularly poor outcome. Third, distinct combinations of co-occurring alterations, such as the nucleoporin 98 (NUP98)–nuclear receptor binding SET domain protein 1 (NSD1) fusion and mutation of fms-related tyrosine kinase 3 (FLT3) or WT1 transcription factor (WT1), were observed, significantly affecting disease outcome. Fourth, alterations in signaling mediators such as N-or K-RAS and the receptor tyrosine kinases KIT and FLT3 appeared to be more prevalent than in adult patients. In contrast, mutations in DNMT3A, IDH1/2, NPM1, or tumor protein p53 (TP53) were less common in pediatric AML. Fourth, some “novel” pediatric-specific chromosomal copy number changes were found, including focal deletions in genes like muscleblind like splicing regulator 1 (MBNL1), zinc finger E-box binding homeobox 2 (ZEB2), E74-like ETS transcription factor 1 (ELF1), or interleukin 9 receptor (IL9R). Collectively, the TARGET AML initiative provided a comprehensive dataset of genetic alterations in pediatric AML that confirmed and extended previous observations indicating that similar to adult patients, pediatric AML is the product of a low number of cooperating mutations frequently involving transcriptional regulators affecting differentiation and self-renewal properties and mutations of signaling mediators (9) (Figure 1). Here, we focused on hallmarks of aggressive pediatric AML fusion oncogenes, including KMT2A, CBFA2T3, and NUP98 fusions.

### Fusion Genes Associated With Aggressive Pediatric AML

The TARGET-AML study suggested that the association of pediatric AML with different fusion oncogenes strongly correlates with age of the patient (8) (Figure 1). Whereas, fusions involving KMT2A, CBFA2T3, or MNX1 are molecular hallmarks of AML affecting infants and early childhood (<3 years), those affecting the core binding factor (RUNX1 and CBFB) or the retinoid acid receptor (RARA) occur at any age but peak in children (3–14 years) or even in young adults (15–39 years). In addition, two particular NUP98 fusions, NUP98–lysine demethylase 5A (KDM5A, a.k.a. JARID1A, or RBP2) and NUP98–NSD1, are molecular hallmarks of cytogenetically silent infant or childhood AML, respectively.

**Figure 2:**
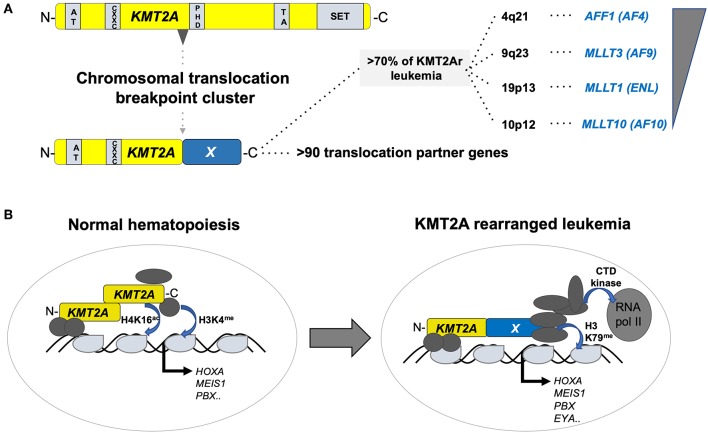
Structure and function of KMT2A fusion oncogenes associated with pediatric leukemia. **(A)** Schematic outline of the KMT2A (a.k.a. MLL1) protein organization containing AT-rich, CXXC, PHD-fingers, transactivation (TA), and a SET methyltransferase domain with recurrent chromosomal breakpoint clusters leading to fusions to >90 different partners, of which >70% are formed by AFF1 (AF4) on 4q21, MLLT3 (AF9) on 9q23, MLLT1 (ENL) on 19p13, and MLLT10 (AF10). **(B)** Simplified illustration of the function of KMT2A in normal hematopoiesis (left) and upon rearrangement in acute leukemia (right). In normal hematopoiesis, KMT2A is proteolytically cleaved into an N- and C-terminal fragment that associated with co-regulators leading to activation of its targets (including, e.g., the *HOX-A* gene cluster, *MEIS1* or *PBX1*) through H4K16 acetylation and H3K4 methylation. In leukemia, KMT2A fusion proteins recruit a super elongation multi-protein complex and the DOT1L H3K79 methyltransferase to activate its target genes including the *HOX-A* gene cluster, *MEIS1, PBX1*, and *EYA*1.

The KMT2A (better known as mixed lineage leukemia, MLL) gene on the long arm of chromosome 11 (11q23) encodes a SET-domain histone methyltransferase that is important for the maintenance of the hematopoietic stem cells (10). KMT2A is the target of chromosomal translocations in adult and pediatric acute leukemia, mostly leading to fusions of the N-terminus of KMT2A with a large number of different partners, of which AF4/FMR2 family member 1 (AFF1, a.k.a. AF4), MLLT3 super elongation complex subunit (MLLT3, a.k.a. AF9), MLLT1 super elongation complex subunit (MLLT1, a.k.a. ENL), and MLLT10 histone lysine methyltransferase DOT1L cofactor (MLLT10, a.k.a. AF10) are the most prevalent (11). Although t(4;11)(q21;q23) leading to a KMT2A–AFF1 fusion is a molecular marker of infant acute lymphoblastic leukemia (ALL), it can occur at any age, and is rarely also found in AML. In contrast, t(9;11)(p22;q23) and ins(10;11)(p12;q23q13) leading to expression of KMT2A–MLLT3 and KMT2A–MLLT10 fusions appear more prevalent in pediatric than in adult AML. Interestingly, KMT2A–MLLT3^+^ disease in infants presents more often as ALL than AML, whereas the phenotype changes into a typical myelo-monocytic AML M5 with increasing age of the patient. The difficulty to classify leukemia with KMT2A fusions, including lymphoid diseases, is also based on the fact that leukemic blasts retain a substantial amount of lineage infidelity and/or plasticity highlighted by frequent co-expression of myeloid markers and relapse of KMT2A^+^ B-ALL as AML (12). Functionally, it is currently thought that KMT2A fusions transform HSPCs by recruitment of a large super elongation protein complex (SEC) that activates transcription of target genes via directly influencing elongation by the RNA polymerase II (RNA-pol II). In addition, KMT2A fusion proteins also recruit the DOT1L histone 3 lysine 79 (H3K79me) methyltransferase that positively regulates expression of critical target genes (13) (Figure 2).

The importance of aberrant transcriptional control is furthermore highlighted by fusions between KMT2A and lysine acetyltransferase 6A (KAT6A a.k.a. Monocytic leukemia zinc finger protein, MOZ, or MYST3) or 6B [KAT6B a.k.a. Moz-related factor (MORF) or MYST4] to the histone acetyl transferases EP300 or CREBBP in some rare cases of pediatric myelodysplastic syndromes (MDS) and AML (14–16). In contrast to adult patients where MYST (MOZ/Ybf2/Sas2/TIP60) fusions are often associated with therapy-related AML, in pediatric patients, these fusions are found in congenital and perinatal leukemia. Interestingly, some infants with KAT2A-CREBBP^+^ AML were reported to go into spontaneous remission; however, the underlying biology remains poorly understood (17, 18).

**Figure 3:**
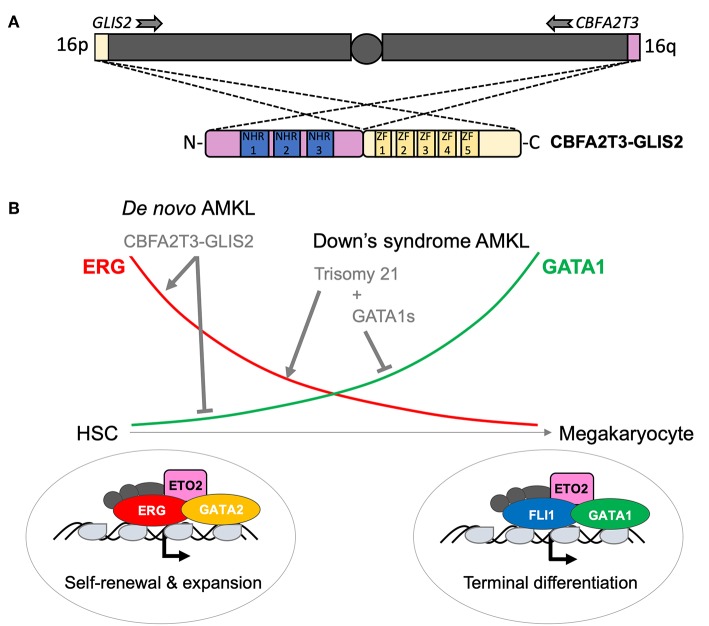
Structure and function of the CBFA2T3–GLIS2 fusion associated with pediatric AMKL. **(A)** Schematic outline of the karyotypically silent inv(16) leading to fusion between the telomerically located GLIS2 (16p13) and CBFA2T3 (16q24) genes, respectively. All known fusions contain the Nervy homology domains (NHR) 1–3 of the transcriptional co-repressor CBFA2T3 and the five zinc fingers (ZF) of the GLIS2 transcription factor. **(B)** Schematic representation of the change in key transcription factors activity during the normal differentiation of hematopoietic stem cells (HSC) toward mature platelet-producing megakaryocytes. ERG and GATA2 activity are higher in HSC and progressively replaced by FLI1 and GATA1 in megakaryocytes. A schematic representation of the consequences of genetic alterations found in pediatric AMKL is also shown. It is currently thought that, while AMKL associated with Down's syndrome target ERG and GATA1 through independent genetic alterations, the CBFA2T3–GLIS2 fusion is able alone to maintain both high ERG and low GATA1 activity contributing to the blockage of differentiation and aberrant self-renewal capacities of AMKL leukemic blasts.

The CBFA2T3 (a.k.a. ETO2 or MTG16) gene on the long arm of chromosome 16, encoding a transcriptional co-repressor, is targeted by two recurrent AML-associated chromosomal rearrangements, the t(16;21)(q24;q22) and the cytogenetically silent inv(16) (p13q24) leading to expression of RUNX1–CBFA2T3 and CBFA2T3–GLIS2 fusions, respectively. Whereas, the first is more prevalent in therapy-related adult AML and rarely found in pediatric patients, the second appears to be an exclusive pediatric lesion (19, 20). NGS strategies allowed the identification of the CBFA2T3–GLIS2 fusion from tumor cells of pediatric patients with *de novo* acute megakaryoblastic leukemia (non-DS AMKL) (21, 22). CBFA2T3–GLIS2 is the most prevalent chromosomal aberration of this disease entity followed by KMT2A, RBM15 (RNA-binding motif protein 15)-MRTFA (Myocardin related transcription factor A) (a.k.a. OTT-MAL), NUP98–KDM5A and other rare events (e.g., GATA2–HOXA9, MN1–FLI1, or NIPBL–HOXA9) (23). CBFA2T3–GLIS2 is not restricted to AMKL but can also be found in cytogenetically normal (CN) pediatric AML with different phenotypes (M0, M1, M2, M4, and M5, according to the FAB classification). Notably, AMKL patients are significantly younger than those with other AML phenotypes (24). Mechanistically, CBFA2T3–GLIS2 binds DNA, through CBFA2T3-associated transcription factors or directly through GLIS2 (GLIS family zinc finger 2) zinc-finger domains at enhancers and regulatory elements and leads to altered transcription and activity of key transcription factors like the upregulation of the ETS transcription factor ERG and a strong downregulation of GATA1 (25). Genetic hijacking of ERG and GATA1 activities represents a common theme among pediatric AMKL as constitutive trisomy 21 (a.k.a. Down's syndrome) AMKL disease progression is characterized by independent genetic alterations also impacting *ERG* (carried by chromosome 21) and *GATA1* (Figure 3) (26, 27). Notably, additional mutations in cohesin components (~50% of patients), CTCF (~20% of patients), epigenetic regulators (~45% of patients), and signaling pathway intermediates (~45% of patients) may also re-enforce an ERG/GATA activity imbalance (28, 29). CBFA2T3–GLIS2 is also associated with aberrant expression of GLI-family target genes, including BMP factors, motivating evaluation of GLI inhibitors' efficacy and specificity for this fusion (21, 22, 30).

**Figure 4:**
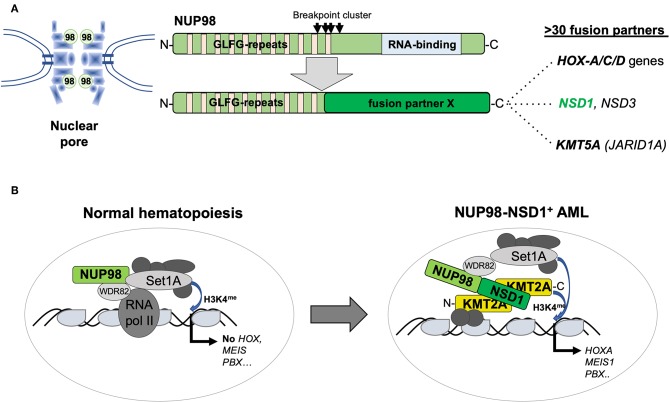
Structure and function of AML-associated NUP98 fusions. **(A)** Schematic outline of the NUP98 pore protein organization containing multiple N-terminal GLFG repeats and a C-terminal RNA binding domain lost by fusion to >30 different fusion partners including homeodomain proteins (*HOXA-C-D* gene cluster) and non-homeodomain proteins including NSD1, NSD3, or KDM5A (JARID1A) [modified from Masetti et al. (30)]. **(B)** Recent work (36) suggested that, in normal cells, in addition to its role as a pore protein, NUP98 is part of a transcriptional co-activator protein complex containing WDR82 and SET1A that regulate their targets (that are not the classical KMT2A targets) by setting H3K4me marks. The NUP98–NSD1 interacts with KMT2A and re-localizes the NUP98-associated WSC activity resulting in activation of classical KMT2A targets including the *HOX-A* gene cluster, *MEIS1* or *PBX1*.

The NUP98 gene on the short arm of chromosome 11 (11p15) encodes a structural component of the nuclear pore but the protein can also function as a transcriptional regulator (31). Similar to KMT2A, NUP98 is targeted by numerous chromosomal translocations or inversions in various but mostly myeloid hematological malignancies, leading to the expression of chimeric proteins containing the N-terminus of NUP98 fused to a large variety of different partners including several homeobox proteins (32). The best studied is t(7;11)(p15;p15), leading to a NUP98–HOXA9 fusion in MDS, chronic myeloid leukemia (CML) in blast crisis, and AML of any age. In contrast, the cytogenetically cryptic t(5;11)(q35;p15) and t(11;15)(p15;q35) translocations, leading to the expression of NUP98–NSD1 or NUP98–KDM5A fusions, respectively, are preferentially found in pediatric AML. NUP98–NSD1 contains the GLFG repeats of NUP98 fused to several PHD finger domains and the SET methyltransferase domain of NSD1 (33). NUP98–NSD1 is one of the most prevalent aberration in pediatric CN-AML, often presenting with a myelomonocytic phenotype (M4/M5; FAB) associated with poor outcome. Interestingly, in the majority of patients, tumor cells also harbor an internal tandem duplication in FLT3 (FLT3-ITD) and/or mutation in WT1 (34). NUP98–KDM5A was identified from a patient with megakaryoblastic leukemia and later shown to be present in about 10% of non-DS-AMKL (35, 36). NUP98–KDM5A contains the GLFG repeats of NUP98 fused to the C-terminal PHD finger domain of the KDM5A histone demethylase. Similar to KMT2A fusions or CBFA2T3–GLIS2, the presence of NUP98–KDM5A confers a poor clinical outcome (23). The mechanism of transformation by NUP98 fusions might involve the N-terminus containing GFLG repeats recruiting a large WDR82-SET1A/B-COMPASS (WSC) protein complex to promote trimethylation of lysine 4 of histone 3 (H3K4me) favoring active transcription (37). On the other hand, the fusion partners appear also to contribute to alter target gene expression such as the HOX-A gene cluster by, e.g., H3K36 methylation (NUP98–NSD1) or by acting as a boundary factor that prevents spreading of repressive polycomb factors (NUP98–KDM5A) (38, 39) (Figure 4).

The MNX1 (a.k.a. HLXB9) gene on the long arm of chromosome 7 (7q36) encodes a homeobox transcription factor that is essential for pancreatic organogenesis, and moto-neuron differentiation, and is involved in a t(7;12)(q36;p13) translocation that is a hallmark of infant AML with poor outcome (8, 40). Although initial reports suggested that this translocation would lead to a chimeric fusion protein containing HLH and ETS domains of ETV6 joined to regulatory sequences and first exon of MNX1 lacking the homeodomain, it appeared that overexpression of MNX1 might be the primary consequence (41). The role of MNX1 in the hematopoietic system remains unclear. However, functional studies suggested that the protein might regulate cell–cell interaction and adhesion of leukemic cells and that aberrant expression of MNX1 leads to differentiation block in megakaryocyte-erythroid progenitor cells (42, 43).

### Signaling Mutations in Pediatric AML

Mutations in signaling mediators such as receptor tyrosine kinases (e.g., FLT3 and KIT) and RAS-related molecules (e.g., N-RAS, K-RAS, PTPN11, or NF1) are found in adult and pediatric AML (8). These mutations generally lead to constitutive activation of interconnected signaling cascades that activate downstream effectors, such as signal transducers and activators of transcription (STAT), ELK, MYC, c-JUN, or NF-kB involved in transcriptional regulation of cell proliferation and survival.

The RAS proteins are a family of highly homologous low-molecular-weight proteins that bind GTP, located at the inner face of the plasma membrane. In the normal situation, the activity of RAS is controlled by hydrolysis of bound GTP by GTPase activating proteins (GAPs) and the replacement of bound GDP with fresh GTP, which is catalyzed by the family of guanine nucleotide exchange factors (GEFs) (44). About 20% of human cancers have activating point mutations of RAS most frequently affecting K-RAS, less N-RAS, and rarely H-RAS (45). The TARGET study reported N/K-RAS mutations in over 40% of investigated pediatric AML cases. Notably, the highest prevalence of RAS mutations was observed in infant patients that also harbored KMT2A fusions (8). Most cancer-associated RAS mutations affect codons 12, 13, and 61 and all compromise the GTPase activity of RAS, preventing GAPs from promoting hydrolysis of GTP on RAS and leading to the accumulation of RAS in the GTP-bound active form. G12D, G12V, G13D, and Q61H are the most prevalent RAS mutations in pediatric AML. Although the prognostic value of RAS mutations is an ongoing matter of debates, like other signaling mutations, they appear to affect the outcome by changing clonal expansion in AML (46).

The FLT3 protein is a class III receptor tyrosine kinase (RTK) family that contains an extra-cellular domain made up of five immunoglobulin-like regions, a single transmembrane region, an intracellular juxtamembrane domain (JMD), and two kinase domains at the carboxyl terminus. Inactivation studies in mice have shown that FLT3 signaling is central to the development of HSPC, B-cells, dendritic cell progenitors, and natural killer cells (47). Binding of FLT3-ligand (FL) leads to dimerization of FLT3 and autophosphorylation of tyrosine residues in the kinase domains, resulting in activation of multiple signaling cascades including RAS/RAF, PI3K/AKT, or STAT5, resulting in increased proliferation and survival. FLT3 is highly expressed in many hematological malignancies and often co-expressed with its ligand FL, suggesting autocrine signaling (48, 49). FLT3 is targeted by two categories of activating mutations, internal tandem duplication (ITD, variable in length) and tyrosine kinase domain (TKD) mutations. FLT3-ITD results in FL-independent dimerization, constitutive phosphorylation, and activation of downstream mediators. FLT3-ITD is found in about 10–20% of newly diagnosed pediatric AML patients and was reported to be an independent prognostic factor for poor outcome particularly for patients with high ITD allelic ratios and/or loss of the wild-type FLT3 allele leading to copy number-neutral ITD homozygosity (48). FLT3-TKD mutations mostly affecting aspartic acid D835 are less common (5–10%) than ITD and seem not to carry the same prognostic significance. Nevertheless, TKD mutation may also occur secondary at relapse of ITD^+^ patients that are treated with FLT3 inhibitors. Notably, FLT3-TKD mutations are particularly prevalent in pediatric leukemia patients with KMT2A fusions (50).

### Mutations in Epigenetic Regulators

In contrast to adult AML, mutations in regulators of DNA methylation and histone modification including Ten-Eleven Translocation2 (TET2), IDH1 or IDH2, Enhancer of Zeste Homolog 2 (EZH2), DNMT3A, and Additional Sex Combs like-1 (ASXL1) are much less prevalent, affecting only about 1–2% of pediatric patients (51). Nevertheless, IDH1 or IDH2 mutations in codons 132 and 140, respectively, were found in over 10% of a cohort of CN pediatric AML mostly in combination with alterations of KMT2A, NUP98, and FLT3-ITD or RAS (52). DNMT methyltransferases (DNMT1, DNMT3A/B), the TET family of enzymes (TET1–3), and IDH1/2 are functionally interconnected (53). DNMTs methylate DNA cytosine residues that can be oxidized from 5-methylcytosine (5mC) to 5-hydroxymethylcytosine (5hmC) by TET enzymes in an iron- and α-ketoglutarate (α-KG)-dependent manner. The presence of an IDH1/2 mutation results in the production of 2-hydroxyglutarate (2-HG), which is structurally very similar to α-KG and competes with α-KG to inhibit α-KG–dependent enzymatic processes (53). Mouse models have shown that inactivation of TET2 and DNMT3A or targeted mutagenesis of IDH1/2 mostly induces preleukemic states and leads to AML development upon cooperation with additional mutations (54–56). Even though rare in pediatric patients, it is important to search for alterations of these factors, as novel approaches for therapeutic targeting (as outlined below) showed very promising results in adult AML.

## Modeling Genetic Lesions in Pediatric AML

Development of recombinant DNA technologies allowed the cloning and characterization of a large number of genetic alterations identified in tumor cells from AML patients. To address their potential for induction and maintenance of a transformed phenotype, the cDNA of a gene of interest carrying a mutation or eventually an entire fusion gene is transferred into hematopoietic cells (cell lines or primary cells) mostly by recombinant retroviruses. Based on the limited access to primary material of a genetically rather heterogeneous disease as pediatric AML, the majority of functional studies are performed in animals, mostly mice. *In vitro* experiments measure the impact of an AML-associated mutation on proliferation and survival but also self-renewal and differentiation of bone marrow (BM)-derived HSPC. The latter two are often determined by measuring the serial replating capacity in growth factor-containing semisolid medium such as methylcellulose (MC). Expression of many KMT2A, NUP98, CBF, or RARA fusions support serial HSPC replating in MC associated with variable blockage of normal maturation. This assay also allows structure-function studies to dissect critical domains of a given gene product. To determine the transforming potential *in vivo*, researchers aim to express the respective cDNA/mini gene in HSPC of their animal model. In one widely used approach, mouse BM-derived cells are virally transduced to overexpress the respective genetic lesion and transplanted into irradiated syngenic recipients [often referred to as BM transplant (BMT) or reconstitution model] (57). Despite the drawbacks of often higher-than-physiological expression, viral integration, and transduction bias of multipotent progenitor cells, this assay allows relatively fast and easy-to-obtain insights into the leukemogenic potential of a given lesion. This strategy was used to show functional cooperation of transcription factor fusions (e.g., KMT2A–MLLT3, KMT2A–MLLT1, NUP98–HOXA9, RUNX1–RUNX1T1, PML–RARA, and others) with signaling mutations like FLT3-ITD, N-/K-RAS, or activating KIT mutations (58, 59). Nowadays, putative leukemogenic driver oncogenes can also be knocked into the mouse genome, resulting in expression regulated from its native promoter/enhancers. In addition, several chemically inducible transgenic mouse lines have been generated that identify the role of a mutation not only for induction but also for maintenance of a leukemic phenotype. Most likely, genome editing using Crispr/Cas9 will facilitate targeted recombination and eventually boost the development of novel AML animal models (60).

As the hematopoietic system of the mouse is not identical to the situation in humans, researchers developed transgenic mouse lines with a humanized hematopoiesis expressing several human hematopoietic regulatory genes in combination with defects of a normal immune response such as the MISTRG strain (61). These humanized mice not only allow to expand patient-derived AML cells but also to study the biology of the disease (62). Immunodeficient mice also allow to model the disease by transplanting human HSPCs engineered to virally overexpress a leukemia-associated fusion oncogene (63) or, more recently, obtained from the hematopoietic differentiation of induced pluripotent stem cells (iPSCs) derived from AML patient blasts (64, 65). Due to space constraints, we focus on some recent findings in mouse models related to those genetic lesions that are recurrently found in pediatric AML.

### Modeling KMT2A Fusion-Driven Pediatric AML

KMT2A fusions, such as KMT2A–MLLT3, are among the best-studied AML-associated alterations. Transgenic knock-in as well as BMT models resulted in a myelo-monocytic AML that closely phenocopied the human disease (66). Strikingly, transduction of lineage-marker-depleted, Kit^+^ Sca1^+^(LSK) cells, common myeloid progenitor (CMP), or granulocyte-macrophage progenitor (GMP) cells with virus expressing KMT2A–MLLT3 or KMT2A–MLLT1 followed by transplantation resulted in a very similar disease (67). To dissect the activity of KMT2A–MLLT3 and KMT2A–MLLT1 in different cells of the hematopoietic hierarchy, we developed DOX-regulated transgenic mouse lines. As expected, we observed that KMT2A–MLLT3 is able to transform hematopoietic stem cells (HSC), but also more committed progenitor (CMP and GMP) cells. However, activation in long-term hematopoietic stem cells (LT-HSC) resulted in some animals in a particularly aggressive AML characterized by high expression levels of the transcription factors MECOM (MDS1 and EVI1 complex locus, a.k.a. EVI1) and ERG, which also characterize human AML with poor outcome (68). In contrast, using the same strategy, we found that activation of KMT2A–MLLT1 preferentially transformed HSC, while CMP were transformed less efficiently and GMP were not transformed at all (69). This finding in transgenic mice might also reflect the situation in patients, as KMT2A–MLLT1 leukemic cells in patients often express lymphoid markers and are diagnosed as ALL or mixed lineage leukemia, whereas KMT2A–MLLT3 mostly present with AML-M5 (11). These observations in adult mice suggest that cells of the hematopoietic hierarchy have a differential sensitivity for a given leukemogenic fusion gene. In attempts to model pediatric KMT2A-rearranged AML, Chen et al. transplanted fetal liver-derived HSPC from a KMT2A–MLLT3 transgenic (knock-in) mice into wild-type mice and observed induction of a leukemic phenotype often expressing lymphoid surface markers after long latency. In contrast, transplantation of adult BM-derived cells led to the typical AML-M5 phenotype (70). Although differential grafting potential of highly cycling fetal liver-derived cells compared to adult BM cells might have influenced these experiments, pediatric but not adult KMT2A–MLLT3^+^ leukemia often express lymphoid markers, strongly suggesting that activation at a particular developmental stage significantly influences disease biology. However, so far, we are not aware of any animal model that appropriately phenocopies KMT2A fusion-driven pediatric AML.

### Modeling NUP98 Fusion-Driven Pediatric AML

Multiple transgenic mouse models have shown that expression of several NUP98 fusions in the hematopoietic system results in various malignancies (32). Functional cooperation between NUP98–HOXA9 and BCR–ABL fusions became a widely used model to study CML in blast crisis (71, 72). Transgenic NUP98–HOXD13 mice, which develop myelodysplasia eventually progressing to AML, are often used to study molecular mechanisms of MDS (73). However, the transforming potential of the preferentially or exclusively pediatric NUP98 fusions (NUP98–KDM5A and NUP98–NSD1) is less clear. Transplantation of adult BM-derived HSPC retrovirally expressing the NUP98–KDM5A fusion resulted in a fully penetrant AML phenotype after 50–100 days, indicating a strong leukemogenic potential. Tumor cells expressed surface markers of early myeloid progenitor cells, but expression of megakaryoblastic markers observed in pediatric cases was not reported (40). Transplantation of adult BM-derived HSPC retrovirally expressing the NUP98–NSD1 fusion was reported to induce an AML phenotype in mice after a long latency with tumor cells expressing myeloid but also early stem cell-related (FLT3, CD34, and KIT) surface markers (38). However, using the same retroviral vector but a slightly different experimental strategy, we only observed development of AML upon co-transduction of NUP98–NSD1 together with FLT3-ITD, a mutation that is found in the majority of the patients (74). Even though these models indicate that NUP98–NSD1 has some transforming potential, alone it seems not sufficient to induce the disease. In addition, most of NUP98–NSD1^+^ AML occurring in children and younger adults present mostly with a myelomonocytic AML for which we currently do not have an appropriate model.

### Modeling Pediatric AMKL Fusion Oncogenes

CBFA2T3–GLIS2, RBM15–MRTFA, NUP98–KDM5A, and KMT2A fusions are found in about 60–70% of non-DS-related pediatric AMKL. Additional rare fusions like GATA2–HOXA9, MN1–FLI1, NIPBL–HOXB9, or NUP98–BPTF were cloned from tumor cells from pediatric AMKL patients (75). Earlier work identified some JAK3 activating mutations in cell lines derived primarily from DS-AMKL, which induced a transient myeloproliferative disease with megakaryoblastic elements when retrovirally expressed in hematopoietic cells from Balb/c mice (76). Knock-in of the MRTFA cDNA at the endogenous Rbm15 locus led to *bona fide* Rbm15–MRTFA fusion expression in mice, altered clonogenic potential of fetal liver-derived hematopoietic cells, and AMKL with a low penetrance. Retroviral co-expression of the thrombopoietin receptor (MPL) carrying an activating mutation (W515L) induced leukemia with morphologic characteristics of AMKL; however, such a combination is rarely seen in patients (77). Using a similar approach to combine a transgenic model of Trisomy 21 (Ts1Rhr) with GATA1s and MPL^W515L^, Malinge et al. demonstrated that all three alterations were required to induce AMKL in mice (78, 79). Retroviral expression of CBFA2T3–GLIS2, GATA2–HOXA9, MN1–FLI1, and NIPBL–HOXB9 allowed serial replating of adult mouse BM-derived hematopoietic cells in MC associated with expression of megakaryocytic markers on some CBFA2T3–GLIS2- and MN1–FLI1-expressing cells. Consistently with the high homology between ERG and FLI1, transplantation of these MN1–FLI1-transduced cells was sufficient for the development of murine leukemia presenting clear features of AMKL (80, 81). However, GATA2–HOXA9- and NIPBL–HOXB9-transduced cells led to penetrant AML phenotypes but with limited megakaryocytic features, and CBFA2T3–GLIS2-transduced cells did not induce any disease (81). Of note, expression of RBM15–MRTFA was also not able to induce leukemia development using a retroviral transduction/BM transplant approach, suggesting that this approach is not suitable to model the leukemogenic activity of all fusion oncogenes (77). Very recent work suggested that transplantation of fetal liver hematopoietic cells retrovirally expressing the CBFA2T3–GLIS2 fusion in lethally irradiated recipients is able to induce an AMKL phenotype co-expressing the CD41 and CD61 megakaryocytic markers but with a limited penetrance (82). Together, these results indicate that better models are required to more faithfully recapitulate pediatric AMKL.

### Challenges to Accurately Model Pediatric Acute Leukemia in Mice

The genetic heterogeneity, as well as the rareness of pediatric AML that limits access to primary cells, urges for the development of models that closely phenocopy the biology of the human disease. Establishment of appropriate models for pediatric leukemia is a challenging task illustrated by the efforts to develop a mouse model for KMT2A–AFF1^+^ infant B cell ALL. The observation that KMT2A fusion^+^ infant leukemias have on average only about two non-silent mutations strongly suggests that the fusion might be sufficient for inducing the disease or that only very few cooperating hits are necessary (8, 83). Expression of a knocked-in KMT2A–AFF1 fusion ORF developed mostly B-cell lymphomas after a long latency in mice (84). An ALL phenotype developing after a relatively long latency was observed in mice carrying a conditional KMT2A–AFF1 allele, or by retroviral co-expression of KMT2A–AFF1 and the reciprocal AFF1–KMT2A fusion (85). More recently, Jim Mulloy et al. demonstrated that transplantation of human CD34^+^ HSPC retrovirally expressing a human–mouse chimeric KMT2A-Aff1 fusion developed pro-B-ALL after a latency of 100–250 days. In contrast, expression of this chimeric fusion in mouse HSPCs resulted in AML, whereas only low titers could be generated of viruses containing the fully human fusion ORF. Although this study was the first that indeed produced a KMT2A–AFF1-driven pro-B-ALL, the disease did not develop in “infant” or newborn mice (86). In another attempt to model KMT2A–AFF1 infant leukemia, Barrett et al. induced the fusion between developmental E12 and E14 to all definitive hematopoietic cells formed during embryonic development using a conditional invertor mouse strain controlled by the VE-Cadherin-cre recombinase. Expression of KMT2A–AFF1 at this early stage increased engraftment and self-renewal of fetal liver cells and provided the cells with a high clonogenic B-lymphoid potential; however, no early progression to B-ALL was observed (87). Interestingly, Menendez et al. earlier found the KMTA2A–AFF1 fusion in BM mesenchymal stoma cells in affected patients, suggesting an early pre-hematopoietic precursor cell origin of the fusion (88). Of note, one cannot exclude that these observations are explained by species-related differences, inappropriate expression levels in cells at a particularly sensitive developmental stage, or the lack of a potential cooperative lesions.

Experiments based on transplantation of fetal liver- or BM-derived cells retrovirally expressing the NUP98–HOXA9 fusion into adult recipients revealed that the age of the cell of origin determines not only the latency period for disease development but also the lineage phenotype and changes of the BM niche (89). In the attempt to model Trisomy 21-associated AMKL, retroviral expression of ERG in murine adult BM leads to 100% of T-cell leukemia, while expression in E12.5 fetal liver cells generated erythro-megakaryocytic leukemia in 40–60% of recipient mice (90). Also, GATA1s and Trisomy 21 have been shown to induce stage-specific alterations of fetal hematopoiesis in both murine transgenic models and humans (91, 92). Proof of concept that neonatal hematological malignancies can also be induced in mice was provided by experiments that modeled juvenile myelomonocytic leukemia (JMML). Hereby, either fetal expression of KrasG12D controlled by Flt3-cre recombinase or constitutive co-deletion of Cbl/Cbl-b resulted in aggressive neonatal myeloproliferative disorders that were lethal within 2–3 weeks after birth (93, 94).

It is very likely that the type of hematopoietic stem or progenitor in which the mutation/fusion first appears is of importance for both development and phenotype of pediatric AML. Although under considerable debates, the normal hematopoietic hierarchy is constituted of a continuum of progenitors presenting different self-renewal and differentiation potential (95, 96). Notably, there is increasing evidence that the fetal and adult hematopoietic hierarchies significantly differ in structure and composition (97). Although not yet demonstrated in murine models of pediatric AML, several AML oncogenes (including KMT2A–MLLT3) studied in an adult context are able to transform both HSC and more committed progenitors (e.g., GMP) while others are not able to do so (67, 98–101). Also, as indicated above, the phenotype of KMT2A–MLLT3^+^ and KMT2A–MLLT1^+^ leukemia was dependent on the stage of the hematopoietic hierarchy in which the driver mutation was expressed (68, 69). Recently, some studies have reported the successful derivation of iPSCs from human AML cells, including from blasts presenting MLL fusions, suggesting that this approach can generate human-based preclinical models (64, 65, 102, 103). Although the frequency of successful iPSC reprograming from AML blasts is likely low, iPSC-derived hematopoiesis followed by transplantation into immunodeficient models may represent a future opportunity to investigate specific stages of the human fetal hematopoietic development that are difficult to access using primary human samples (104).

**Figure 5:**
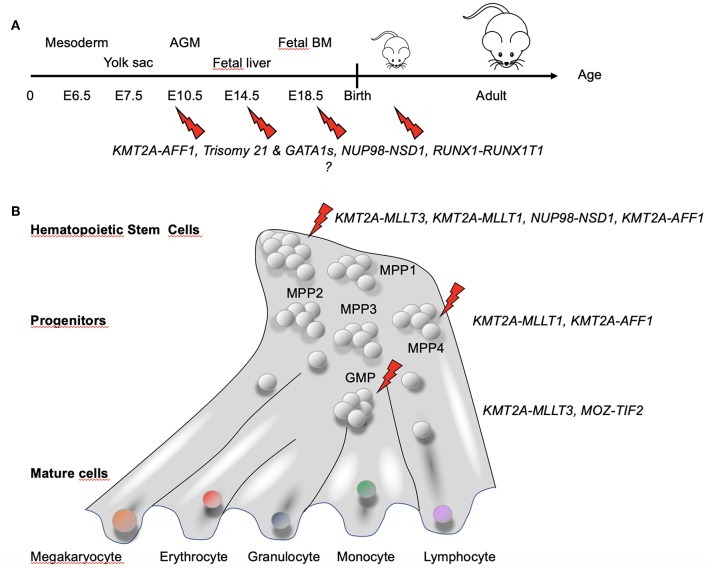
Hematopoietic developmental stage and hierarchy-dependent susceptibility for transformation by AML-associated fusion oncogenes. **(A)** Increasing evidence suggests that pediatric AML-associated oncogenes have a particular window of opportunity during development to transform hematopoietic cells. GATA1s and Trisomy 21 have been shown to induce stage-specific alteration of fetal hematopoiesis (86). For KMT2A–AFF1^+^ leukemia, the fusion was found not only in hematopoietic precursors but also in BM stroma cells (83), suggesting that the fusion might target a very early precursor cell that maintain some mesenchymal properties. **(B)** Differential susceptibility of cells of the hematopoietic hierarchy for transformation by fusion oncogenes associated with pediatric AML [adapted from Rodriguez-Fraticelli et al. (95)]. Together, these observations suggest that both the developmental stage and the type of cell in the hematopoietic hierarchy in which a genetic alteration occurs determines whether a leukemia will develop and the associated disease phenotype and aggressiveness.

Collectively, these studies indicated several factors to be taken into account for appropriately modeling pediatric AML in mice: (1) driver (and eventually also cooperating) mutations most likely need to be active *in utero*; (2) a driver and cooperating lesion might not occur and/or be active at the same developmental stage; (3) a given genetic lesion (e.g., fusion gene) has its optimal expression level; and (4) particular developmental cell stages or identity are likely more permissive to transformation than others (Figure 5).

## Molecular Targeting of Pediatric AML

**Figure 6:**
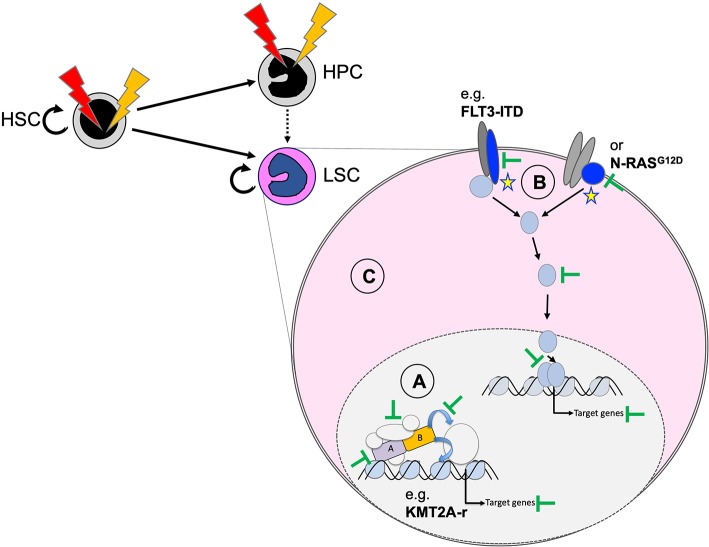
Potential therapeutic targeting of functionally cooperating molecular mechanisms. Personalized efficient AML therapies will most likely be based on a combination of strategies that target the driver mutation **(A)**, cooperating processes **(B)**, and general characteristics of the transformed state **(C)**. Targeting the driver can be achieved by degradation of the fusion oncogene (e.g., as shown for PML–RARA by ATRA and arsenic) and blocking critical protein–protein interactions (e.g., MENIN–KMT2A interaction for chromosome binding of KMT2A fusions), related enzymatic activities, or essential downstream targets. Cooperating mutations, including constitutive active protein tyrosine kinases or RAS proteins, can be targeted with highly selective and potent small-molecule inhibitors (e.g., FLT3 inhibitors). The transformed state can be impaired by blocking survival by primed BH3-apoptosis regulators (e.g., BCL2, MCL1) or by targeting altered metabolism-related regulators (e.g., mutated IDH1/2).

Functional cooperation studies in cellular and animal models suggest that we can group AML-associated mutations into drivers that are essential for induction and maintenance of the disease, and cooperating mutations that support expansion of the malignant clone or may facilitate transformation by mostly metabolic modifications (101). As a consequence, inactivation or degradation of the driver might represent the most promising approach for long-term cure of the disease. However, in cases without a clearly defined driver, inhibition of other cooperating lesions (such as constitutive active protein kinases) or interfering with more general dependencies of transformed hematopoietic cells might provide an alternative strategy (Figure 6). Here, we discuss selected targeting strategies for pediatric AML that are either effective in the clinic, being explored in ongoing trials or just demonstrated as proof of concept in preclinical models.

### Targeting the Driver Mutation (e.g., Fusion Oncogene)

Positive proof of concept that long-term cure can be achieved in AML patients through inactivation of the driver mutation comes from acute promyelocytic leukemia (APL) that is, in the vast majority of the cases, driven by the PML–RARA fusion resulting from t(15;17)(q22;q21). Although, the TARGET pediatric AML study did not report PML–RARA^+^ APL patients, who are often considered separately for therapeutic reasons, they make up to of 5–10% of pediatric AML patients in the United States (105). Notably, about 10% of the pediatric cases clinically presenting as APL seem not to carry any RARA fusion (106). Several transgenic mouse models have shown that the PML–RARA fusion is essential but most likely not sufficient to induce the disease as it cooperated with other mutations such as FLT3-ITD (107). Pioneer work by Hughes de Thé and Zhu Chen demonstrated that pharmacological doses of all trans retinoid acid (ATRA) or arsenic trioxide (As_2_O_3_) induce different molecular mechanisms that ultimately lead to proteasome-dependent degradation of the PML–RARA fusion protein (108). A phase III randomized multicenter trial demonstrated clinical efficacy and superiority of the combination of ATRA with As_2_O_3_ over ATRA and chemotherapy in adult patients (109). Pediatric APL patients treated with ATRA and anthracyclines or As_2_O_3_ reached an estimated overall 5- and 8-year survival of >95% (106). Collectively, these observations strongly suggest that targeted degradation of a driver fusion oncogene is the strategy of choice for long-term cure of a significant fraction of AML patients.

Multiple mouse models demonstrated that several KMT2A fusions are strong leukemogenic oncogenes that drive the disease in the presence of few, if any, cooperating mutations (66, 110). We were able to show that induction and maintenance of a transformed state of murine hematopoietic cells by the KMT2A–MLLT3 or KMT2A–MLLT1 is dependent on the fusion dose and is fully reversible (68, 69), indicating that targeted reduction of the fusion protein might be sufficient to induce differentiation and dissolve the leukemic phenotype. Biochemical studies suggested that leukemogenic KMT2A fusions form large protein complexes that bind to and activate KMT2A targets in an uncontrolled manner (111). Recent experimental work suggests that KMT2A fusion-mediated transformation could be impaired by stabilization of the non-rearranged protein, which naturally undergoes proteasomal degradation regulated by casein-kinase II and IRAK4-dependent phosphorylation events (112, 113). The transforming potential of KMT2A fusions depends on several protein–protein interactions as well as enzymatic activities that have the potential for therapeutic interference. Interaction of the N-terminus of KMT2A with MENIN and the adapter protein LEDGF is essential to bind to critical targets and productive transformation by KMT2A fusion genes (114). Structure-function studies identified critical interphases, and small molecules were developed that disrupted binding of MENIN to KMT2A and impaired KMT2A fusion transformation (115–117). Constant optimization allowed the generation of MENIN protein–protein interaction (PPI) inhibitors that impair the viability of KMT2A fusion-driven cells at a nanomolar concentration (118). Notably, MENIN PPI inhibitors were shown to have potent anti-cancer activity not only in KMT2A fusion-driven leukemia but also in prostate tumors (119, 120). Novel, more potent and stable MENIN PPIs with increased selectivity for KMT2A fusions have been presented at ASH 2018 with potent anti-leukemic activity in patient-derived xenotransplant (PDX) models of AML (121).

KMT2A fusion complexes recruit the DOT1L histone H3 lysine 79 (H3K79) methyltransferase that positively regulates expression of the target genes most likely by preventing association of the sirtuin-1 (SIRT1) deacetylase complex resulting in loss of H3K9 and H4K16 acetylation, and reduced SUV39-mediated methylation on H3K9m3 (122). The maintenance of a KMT2A fusion-induced transformed state showed high dependence of DOT1L-mediated H3K79 methylation and small-molecule DOT1L inhibitors showed promising anti-tumor activity *in vitro* and *in vivo* in preclinical models (123). However, although application of a DOT1L inhibitor (Pinometostat, a.k.a. EPZ-5676) reduced H3K79 methylation, only modest clinical activity was observed in adult patients with KMT2A rearranged leukemia (124).

In addition to MENIN/LEDGF or DOT1L, targeted interference with other components of the KMT2A fusion complexes (e.g., WDR5, BRD4, CDK9) have been explored in various preclinical models (125). Interestingly, AML cells carrying CEBPA mutations leading to expression of the short oncogenic CEBP/α p30 isoform appear sensitive to pharmacological targeting of the KMT2A complex (126). However, so far, no strategy has been reported that allows selective degradation of KMT2A fusion proteins to replicate the success in APL.

Studies with Drosophila cells showed that NUP98 acts as a transcriptional activator physically interacting with non-specific lethal (NSL) and Trithorax (KMT2A) protein complexes (127). More recent work suggested that NUP98 and some NUP98 fusion proteins (NUP98–HOXA9, NUP98–NSD1, and NUP98–HOXD13) physically interact with proteins of the human NSL and KMT2A complexes such as WDR5 or MOF most likely through the GLFG repeats (37, 128). Notably, conditional genetic ablation of KMT2A significantly reduced the leukemogenic activity of the NUP98–HOXA9 fusion *in vivo*. In addition, KMT2A-dependent gene expression signatures from murine NUP98–HOXA9 transformed cells resembled human NUP98–NSD1-derived profiles, suggesting at least overlapping pathways. These observations suggest that therapeutic targeting of KMT2A would also be effective against NUP98 fusion-driven AML. However, more translational studies found significant differences in gene expression signatures of pediatric AML cases harboring either KMT2A fusions or NUP98–NSD1. Indeed, whereas the first group is often characterized by increased expression of EVI1, the latter is associated with increased expression of another PRDM family member PRDM16 (a.k.a. MEL1) (32, 129, 130). Nevertheless, the activity of leukemogenic NUP98 fusions might be impaired by blocking the activities of distinct partner genes. Leukemic blasts immortalized by retroviral expression of the NUP98–NSD1 fusion showed elevated levels of H3K36me marks on putative target genes including the *Hox-A* gene clusters leading to the idea that small molecules blocking the NSD1 SET methyltransferase domain might have anti-leukemic activity (38, 131). Interestingly, disulfiram (DSF), known for its aldehyde dehydrogenase blocking activity, was found to induce apoptosis of murine myeloblasts transformed by PHD-containing NUP98–KDM5A and NUP98–PHF23 fusions. Although the detailed molecular mechanism remains unclear, it appeared that DSF blocked the interaction of the fusion proteins with the promoters of critical downstream targets like *HoxA7-10, Meis1*, and *HoxB5* (132).

Although the use of high-dose cytarabine has improved the outcome of core binding factor (CBF) AML, we still lack efficient strategies to selectively inactivate the RUNX1–RUNX1 partner transcriptional co-repressor 1 (RUNX1T1, a.k.a. CBFA2T1 or ETO) or CBFB–MYH11 driver fusions. Earlier work found that oligomerization of RUNX1–RUNX1T1 through the nervy homology 2 (NHR2) domain of RUNX1T1 was essential for its activity as a transcriptional corepressor and mediator of self-renewal to BM cells (133). Interfering with tetramerization by peptides and by small molecules reduced the oncogenic activity of the RUNX1–RUNXT1 fusion in preclinical models (134, 135). RUNX1–RUNXT1 seems to form stable complexes containing hematopoietic co-factors including E-proteins, of which the interaction, e.g., between the NHR2 domain of RUNX1T1, with a novel binding motif in E proteins seems critical (136). The oncogenic potential of the RUNX1–RUNXT1 fusion was also shown to depend on EP300-medicated acetylation of distinct lysine (K24, K43) lysine residues; hence, blocking EP300 activity impaired leukemic transformation (137). Pioneer work by Illendula et al. provided a novel concept to molecularly target CBFB–MYH11^+^ AML. They developed a small molecule (AI-10-49) that selectively binds the CBFB–MYH11 fusion protein, resulting in RUNX1-mediated repression of the potent oncogenic driver MYC (138, 139). CBFA2T3 is highly homologous to RUNX1T1 containing nervy homology domains (NHR1–3) that mediate oligomerization of the AMKL-associated CBFA2T3–GLIS2 fusion. Notably, overexpression of a small NHR2 peptide (NC128) was able to significantly reduce the leukemia development of a CBFA2T3–GLIS2^+^ human AMKL cell line in immunodeficient mice (25). Collectively, the oncogenic activity of CBF-related fusions can be impaired by interfering with oligomerization (RUNX1–RUNX1T1 and CBFA2T3–GLIS2) and post-translational modification (RUNX1–RUNXT1) or by impairing binding to RUNX1 (CBFB–MYH11); however, no such strategies were so far successfully translated into the clinic.

### Targeting Cooperating Mutations (e.g., Tyrosine Kinase Mutation)

Several small molecules have been established that block the uncontrolled activity of FLT3-ITD, ranging from pan-kinase inhibitors like Sunitinib, to promiscuous inhibitors of multiple tyrosine kinases including Sorafenib, Midostaurin (a.k.a. PKC412), or Lestaurtinib (a.k.a. CEP-701), to very selective compounds such as Quizartinib (a.k.a. AC220), Tandutinib (a.k.a. MLN518), Crenolanib, or Gliternitinib (a.k.a. ASP2215) (140). Based on an international randomized controlled study showing that the combination of Midostaurin and chemotherapy improved the outcome of adult AML patients, the drug recently became FDA-approved for therapy of *de novo* FLT3-mutated AML (141). Very recently, Gliternitinib was also FDA-approved for relapsed/refractory AML with FLT3 mutations based on results from the ADMIRAL trial (142). Several FLT3 inhibitors have been explored in small clinical trials in pediatric AML patients, and partial or complete responses were reported not only in KMT2A-rearranged ALL (Midostaurin, Lestauritinib), but also in refractory/relapse AML (Sorafenib) (143). Promising results have been reported with the combination of the selective FLT3 inhibitor Quizartinib with chemotherapy in children with relapse or refractory AML or KMT2A-rearranged ALL (144). However, more selective FLT3 inhibition has been linked to resistance mutations in FLT3-ITD^+^ AML particularly affecting the gatekeeper (F69L) or activation loop (D835/I836) residues (145). Future prospective controlled clinical studies will be necessary to show the profit and risks of selective FLT3 inhibition in pediatric AML.

Based on *in vitro* and *in vivo* cooperation in AML, targeted inhibition of constitutive active mutant RAS should be of therapeutic benefit. Although long viewed to be undruggable, recent observations suggest that pharmacological inhibition of RAS could be achieved (146, 147). Earlier targeting attempts focused on interfering with RAS posttranslational modification and farnesylation of the CAAX motif necessary for localization of the protein to the cellular membrane. Small-molecule farnesyltransferase inhibitors exhibited anti-leukemic activity in H-RAS but not in K-RAS mutant AML. However, the addition of Tipifarnib (Zarnestra), a selective non-peptidomimetic competitive farnesyltransferase inhibitor, to low-dose cytarabine did not improve outcome in older AML patients (148). More recent work provided proof of concept of targeted interference with distinct RAS mutations: compounds were identified that block K-RAS^G12C^ by forming covalent disulfide bridges with the cysteine. Other compounds were found to block GDP-bound K-RAS^G12C^ and selectively impair cancer growth. Impairing RAS activity by blocking the interaction with downstream mediators, genetic depletion by anti-sense oligos, or interfering with RAS dimerization also suggested that pharmacological targeting could be achieved. However, clinical translation of targeting mutated RAS in cancer has not yet been achieved.

In contrast to adult AML, mutations in the genes encoding for IDH1 and IDH2 are rare in pediatric AML (51, 52). However, small molecules were generated that potently and selectively inhibit mutant IDH1 or IDH2 through binding in an allosteric manner at the interface of the dimerized enzymes (149, 150). IDH1^R132H/C^- or IDH2^R140Q^-selective inhibitors were shown to induce differentiation of primary AML cells *in vitro* and in PDX models, leading to a statistically significant survival benefit (151). Phase I/II clinical trials with Ivosidenib (targeting IDH1-R132) and Evasidenib (targeting IDH2^R140Q^) in refractory or relapsed adult AML patients show overall response rates >40% with about 20% complete remission over several months underlining the potential for these compounds for personalized therapeutic strategies (152, 153). However, some patients developed clinical resistance to Evasidenib by secondary mutations in trans, in the IDH2 allele without the neomorphic R140Q mutation (154). Despite these limitations, the fact that IDH1/2 mutations can be selectively blocked by clinically effective small-molecule inhibitors urges for clinical trials in pediatric AML patients.

### Targeting Hallmarks of Transformed Cells and the Immune System

It is well-known that malignant transformation leads to various cellular dependencies that may offer targets for therapeutic intervention (155). A very promising emerging strategy is to interfere with the cells' capability to evade programmed cell death known as apoptosis. In a simplified view of this complex regulatory pathway, apoptotic cell death is prevented by pro-survival BCL2-like proteins (e.g., BCL2, BCL2L1: a.k.a. BCL-XL, MCL1) by keeping in check the cell death effector proteins BAX and BAK that are activated by BH3-only proteins (e.g., BCL2L11: a.k.a. BIM; BBC3: a.k.a. PUMA, BAD, BID; PMAIP1: a.k.a. NOXA) (156). Small molecules (e.g., Venetoclax, a.k.a. ABT-199) that mimic the function of BH3-only proteins (“BH3-mimetics”) have been developed that are inducing apoptosis not only in lymphoid neoplasms like CLL or B-cell non-Hodgkin's lymphoma but also in myeloid neoplasms including AML (157, 158). Selective BCL2 inhibition by Venetoclax induces rapid cell death in AML cells with an IC_50_ as low as 10 nmol/L (159). A phase II study revealed that Venetoclax monotherapy has potent anti-leukemic activity in high-risk relapsed/refractory adult AML patients (160). Small-molecule MCL1 inhibitors (e.g., VU661013, AMG176) were shown to be synergistic and rescued Venetoclax resistance of AML cells (161, 162). In addition, Venetoclax showed synergistic therapeutic activity in combination with other drugs including low-dose cytarabine, JAK1/2 inhibitors, or DNMT1 inhibitors (decitabine, azacytidine) (163–165). Moreover, inhibition of BCL2 was found to enhance the anti-leukemic activity of FLT3 inhibitors (Midostaurin, Gliternitinib) in preclinical AML models (166). Very recent work suggested that TP53, the apoptotic signaling network, and the mitochondrial functionality are the drivers of Venetoclax sensitivity in AML cells (167). These observations in adult AML patients initiated some studies to explore Venetoclax in pediatric patients with relapse/refractory malignancies including acute leukemia (NCT03236857) (168).

Intensive research is currently ongoing that aims to therapeutically target the capacity of pediatric leukemia cells to escape destruction by the immune system (169). Some studies reported some significant therapeutic responses in AML treated with antibody-drug conjugates (ADCs) targeting surface molecules like Gemtuzumab, a calicheamicin-conjugated antibody against CD33 and the response seems to correlate with a splicing polymorphism affecting the antibodies' binding site (170). Potent anti-leukemic activity has been reported for bispecific T-cell engaging antibodies (BiTEs) targeting T-cell CD3 and CD19 (Blinatumomab) on B-cell ALL cells leading to FDA approval to treat pediatric B-ALL (171). Several BiTEs targeting some AML-associated surface proteins (CD33, CD123, and CD371) that have shown potent experimental activities are currently undergoing clinical trials (172). The immunotherapy revolution in pediatric hematologic cancers is mostly marked by the development of chimeric antigen receptor T-cells (CAR-T). Remarkable success was reported by targeting CD19 on relapsed/refractory B-ALL patients (173). CAR-T approaches to target AML-associated antigens (CD33 and CD123) have been explored in preclinical models, but it appeared that yet to be defined more tumor cell-selective epitopes might be necessary to reach the efficacy observed in B-ALL (174). Very recent experimental studies suggested improved anti CD33 CAR-T therapy for AML by genome editing-mediated ablation of CD33 in HSC (175, 176). Finally, the success of checkpoint inhibitors mostly antibodies targeting immune suppressive antigens such as PD-1, PD-L1, or CTLA-4 in some solid tumors associated with a high mutational burden such as malignant melanoma initiated intensive study for their potential in hematological malignancies including AML (177). PD-1 and/or PD-L1 are expressed in AML cells, and their blockade coupled with depletion of regulatory T-cells showed potent anti-leukemic activity in preclinical models (178). Several monoclonal antibodies (e.g., Nivolumab, Prembrolizumab, Durvalumab, and Ipilimumab) are currently studied for their anti-leukemic potential in refractor/relapse AML patients; however, checkpoint inhibitors alone seem to be much less effective in AML than in solid cancers (179).

## Author Contributions

Both authors have made a substantial, direct and intellectual contribution to the work, and approved it for publication.

### Conflict of Interest

The authors declare that the research was conducted in the absence of any commercial or financial relationships that could be construed as a potential conflict of interest.
