# Corrigendum: Pediatric Acute Myeloid Leukemia (AML): From Genes to Models Toward Targeted Therapeutic Intervention

**DOI:** 10.3389/fped.2019.00466

**Published:** 2019-11-13

**Authors:** Thomas Mercher, Juerg Schwaller

**Affiliations:** ^1^INSERM U1170, Equipe Labellisée Ligue Contre le Cancer, Gustave Roussy Institute, Université Paris Diderot, Université Paris-Sud, Villejuif, France; ^2^Department of Biomedicine, University Children's Hospital Beider Basel (UKBB), University of Basel, Basel, Switzerland

**Keywords:** pediatric AML, genomic landscape, mouse models, fusion oncogene, therapeutic targeting, UKBB

In the original article, there was a mistake in the legend for **Figure 5** as published.

In the legend of **Figure 5**, it states “[adapted from Carmichael et al. (90)]” instead of “[adapted from Rodriguez-Fraticelli et al. (95)]”. The correct legend appears below.

**Figure 5** Hematopoietic developmental stage and hierarchy-dependent susceptibility for transformation by AML-associated fusion oncogenes. **(A)** Increasing evidence suggests that pediatric AML-associated oncogenes have a particular window of opportunity during development to transform hematopoietic cells. GATA1s and Trisomy 21 have been shown to induce stage-specific alteration of fetal hematopoiesis (86). For KMT2A–AFF1^+^ leukemia, the fusion was found not only in hematopoietic precursors but also in BM stroma cells (83), suggesting that the fusion might target a very early precursor cell that maintain some mesenchymal properties. **(B)** Differential susceptibility of cells of the hematopoietic hierarchy for transformation by fusion oncogenes associated with pediatric AML [adapted from Rodriguez-Fraticelli et al. (95)]. Together, these observations suggest that both the developmental stage and the type of cell in the hematopoietic hierarchy in which a genetic alteration occurs determines whether a leukemia will develop and the associated disease phenotype and aggressiveness.

The authors apologize for this error and state that this does not change the scientific conclusions of the article in any way. The original article has been updated.

